# Restoring the Duality between Principal Components of a Distance Matrix and Linear Combinations of Predictors, with Application to Studies of the Microbiome

**DOI:** 10.1371/journal.pone.0168131

**Published:** 2017-01-13

**Authors:** Glen A. Satten, Robert E. Tyx, Angel J. Rivera, Stephen Stanfill

**Affiliations:** 1 Division of Reproductive Health, National Center for Chronic Disease Prevention and Health Promotion, Centers for Disease Control and Prevention, Atlanta, GA, United States of America; 2 Division of Laboratory Sciences, National Center for Environmental Health, Centers for Disease Control and Prevention, Atlanta, GA, United States of America; National Institute of Environmental Health Sciences, UNITED STATES

## Abstract

Appreciation of the importance of the microbiome is increasing, as sequencing technology has made it possible to ascertain the microbial content of a variety of samples. Studies that sequence the 16S rRNA gene, ubiquitous in and nearly exclusive to bacteria, have proliferated in the medical literature. After sequences are binned into operational taxonomic units (OTUs) or species, data from these studies are summarized in a data matrix with the observed counts from each OTU for each sample. Analysis often reduces these data further to a matrix of pairwise distances or dissimilarities; plotting the first two or three principal components (PCs) of this distance matrix often reveals meaningful groupings in the data. However, once the distance matrix is calculated, it is no longer clear which OTUs or species are important to the observed clustering; further, the PCs are hard to interpret and cannot be calculated for subsequent observations. We show how to construct approximate decompositions of the data matrix that pair PCs with linear combinations of OTU or species frequencies, and show how these decompositions can be used to construct biplots, select important OTUs and partition the variability in the data matrix into contributions corresponding to PCs of an arbitrary distance or dissimilarity matrix. To illustrate our approach, we conduct an analysis of the bacteria found in 45 smokeless tobacco samples.

## Introduction

Advances in sequencing technology have revolutionized our view of the microbiome, the microbial communities that exist in almost every environment including within humans and other animals. In the past, study of the microbiome was limited to what grows in culture. The advent of sequencing studies has removed this restriction. By sequencing the 16S rRNA gene, present in all bacteria and almost exclusive to bacteria, it is possible to survey the bacterial composition of samples irrespective of whether they grow easily in culture. The large number of sequences obtained by modern genotyping methods means that bacteria present at very low prevalence can be observed. The resulting data on bacterial abundance are highly complex and analyses often require dimension reduction before important features can be found (in a microbiome study, the OTU counts or frequencies play the role of ‘features’ in a general machine learning context).

In a microbiome study, sequences are typically grouped into operational taxonomic units (OTUs) based on similarity using a bioinformatic pipeline such as QIIME [1] or Mothur [2]. These pipelines produce OTU counts (abundances) that can be summarized in a data matrix *X*; here we take the rows to correspond to observations and the columns to species or OTUs. Since in a microbiome experiment the number of species or OTUs will typically far exceed the number of observations, some sort of dimension reduction is required. As with other studies in ecology, it is common practice to use the species (OTU) abundance data in *X* to calculate a distance or dissimilarity matrix Δ with Δ_*ij*_ denoting the distance between the *i*th and *j*th observation. The distance matrix can be a highly nonlinear function of the data in *X* and may in fact require external data for calculation. For example, the Unifrac distance [3, 4], commonly used in microbiome studies, is a functional of the phylogenetic tree that summarizes the genetic distance between the OTUs, and thus requires genetic sequence data to calculate. Here we do not distinguish between dissimilarity measures that are or are not distance metrics, and generically refer to all dissimilarities as ‘distances.’

The distance measures used by Ecologists (see [5] for an exhaustive discussion) are often very successful at describing the observations in the sense that the first few principal components (PCs) of the (appropriately centered and scaled) distance matrix allow visual separation of the data into meaningful groups. While this separation is useful in showing that OTUs vary systematically across groups, investigators often wish to know which OTUs contribute most to this separation. However, once a distance is calculated, it is difficult to know which species or OTUs contribute to the observed distances, or to place future observations in an ordination plot to see if they cluster with the ‘correct’ group.

In high-dimensional data, important linear combinations of features are frequently obtained by calculating the PCs of *X*^*T*^ *X*, the correlation or covariance matrix of the data, depending on how *X* is scaled. These PCs can also be obtained from a singular value decomposition (SVD) of the data matrix *X*, which yields a set of singular vectors for observations and a set of singular vectors for features (here, OTUs or species). This approach has the advantage that there is a ‘duality’ between the two sets of singular vectors, so that if one set of vectors is known, the other set can be immediately obtained. This duality has useful consequences; the ‘factor loadings’ (coefficients of the corresponding singular vector in feature space) can be obtained for each component in observation space to see which features contribute most, or a biplot can be constructed. In addition, the singular vectors in observation space can be used as predictors in a model, because the duality assures we can interpret and calculate them for future observations. However, the cost is that we are implicitly using *XX*^*T*^ to measure similarity, since the singular vectors for observations are eigenvectors of *XX*^*T*^. The goal of this paper is to restore the duality between the set of eigenvectors for an arbitrary choice of distance matrix Δ, and a set of vectors in feature space, to the largest extent possible.

A motivating example is an analysis of the bacteria found in 45 samples from three types of smokeless tobacco products (dry, moist, and brown toombak) reported elsewhere [6]. Using sequence data from the V4 region of the 16S rRNA gene, we used the QIIME pipeline [1] to categorize the 3,738,578 observed sequences into 5345 OTUs. After applying a thresholding criterion [6], we reduced the number of OTUs to 271 while retaining 3,555,575 (95%) sequences. Tyx et al. [6] found that the first three principal components of the (weighted) Unifrac distance matrix were very successful at differentiating the tobacco types, while also showing that replicates of the same product were closely clustered. However, we cannot know which OTUs are influential in this result. Further, we are unable to use the OTU frequencies of subsequent samples to see if their predicted type (as determined by their placement on the plot of PCs) are consistent with our original analysis. Finally, we cannot make a biplot that uses the ordination obtained using the UniFrac PCs to visualize which OTUs are influential in predicting tobacco type.

The approach we take here is to construct approximate decompositions of the data matrix that mimic the SVD. We first recall how the singular value decomposition (SVD) ensures a connection between eigenvectors of observations and OTUs when the data matrix is decomposed using a SVD, and then present approximate SVD-like decompositions that use the eigenvectors of an arbitrary distance matrix such as the Bray-Curtis or UniFrac distance in the role of the singular vectors for observations. We then show how these SVD-like decompositions can be used to partition the total sum of squares in the data, to aid in choosing the number of components to use and to determine the amount of variability explained by each OTU. In the results, we analyze the tobacco bacteria data to evaluate the performance of the methods we are proposing. We then discuss rarefaction and a kind of weighted analysis that connects two of the approaches we consider. Finally we conclude with a brief discussion.

## Duality between a Distance Matrix and Linear Combinations of OTUs

### Duality and the Singular Value Decomposition

Data from a 16S rRNA microbiome experiment can be summarized in a *n* × *p*-dimensional data matrix *X* where *n* is the number of observations and *p* is the number of species or OTUs. The elements of *X* count the number of reads in observation *i* that fall into OTU *j*. The row sums, referred to as the library size, are thought to be largely ancillary; thus, the count data in *X* is often converted to OTU frequencies by dividing the counts in each row by the corresponding library size (to put each row on the same scale) and then data for each OTU is centered by subtracting the mean OTU frequency. An interesting property of count data scaled and centered in this way is that both row and column sums are zero. Whatever scaling and centering is applied, the data matrix *X* can always be written using the singular value decomposition (SVD) as

$$\begin{array}{c} X=L\Sigma R^{T} \end{array}$$ (1)

where *L* is a *n* × *q* matrix with orthonormal columns, Σ is a *q* × *q* diagonal matrix having positive entries, and *R* is a *p* × *q* matrix with orthonormal columns, where *q* is rank of *X*. If we are willing to measure similarity between observations using Δ = *XX*^*T*^, then the columns of *L* comprise the coordinates of the observations in a principal components analysis, or a principal coordinates analysis (PCoA) if count data in *X* have been scaled and centered as described above, since the columns of *L* are also the principal components of *XX*^*T*^. Equivalently, we can first calculate the PCs of *X*^*T*^ *X* to obtain *R*, the PCs of the covariance (or correlation, depending on scaling) matrix of OTUs. Ecologists refer to the representation of observations by coordinates in a low-dimensional (typically in 2 or 3) space as ordination. The SVD is also the starting point for constructing a biplot of the data.

If the data from each observation is standardized and we use Δ = *I* − *XX*^*T*^ to measure distance then using Eq (1) we see that the eigenvectors of Δ are given by the columns of *L*. In this situation, given only the *k*th PC of Δ (i.e., *L*_*k*_, the *k*th column of *L*) we could use Eq (1) to obtain the ‘factor loadings’ *R*_*k*_ (i.e., the *k*th column of *R*) by rewriting Eq (1) as

$$\begin{array}{c} \Sigma_{kk}R_{k}=L_{k}^{T}X\;\;\text{.} \end{array}$$ (2)

The constant of proportionality (Σ_*kk*_) can be determined by normalizing *R*_*k*_. The factor loadings from Eq (2) contain information on which OTUs are important predictors of the *k*th PC. Conversely, given the matrix of factor loadings *R* and diagonal matrix of constants of proportionality Σ, the eigenvectors of Δ (i.e., the columns of *L*) could be reconstructed by rewriting Eq (1) as

$$\begin{array}{c} L=XR\Sigma^{-1}\;\text{.} \end{array}$$ (3)

Representation Eq (3) allows us to use observed OTU frequencies for a new observation to see where it falls in an ordination plot of existing data. Of course, (Eqs (2) and (3)) are immediate consequences of the SVD and coordinates for observations *L*, factor loadings *R* and constants Σ can be calculated simultaneously.

If we wish to use an arbitrary distance matrix Δ, then the eigenvectors of Δ will not correspond to the left singular vectors of *X*. As a result, Eq (2) cannot be used to express the eigenvectors of Δ as linear combinations of OTU frequencies and Eq (3) cannot be used to determine the PCs of a new observation. Because Δ is real and symmetric, we can always write Δ = *BEB*^*T*^ where *B* is orthogonal and *E* is diagonal; however, the elements of *E* may not all be positive unless Δ is Euclidean.

We can attempt to restore the relationship between the eigenvectors of Δ and linear combinations of the rows of *X* in two ways, either using the singular value decomposition of *X* as our guide, or using prediction of the left singular vectors of *X* (that are used for ordination) as our guide. In the first case, we can seek a decomposition of *X* that looks like the SVD, but uses *B* in place of the left singular vectors. Specifically, we can seek a matrix *V* with normalized columns and a diagonal matrix *D* with nonnegative elements that minimize the objective function

$$\begin{array}{c} f_{d}\left(V,D\right)=\;||X-BDV^{T}{\left|\right|}_{F}^{2} \end{array}$$ (4)

where ${\left||M|\right|}_{F}^{2}=Tr\left(M^{T}M\right)=\sum_{i,j}M_{ij}^{2}$ is the Frobenius matrix norm used for least-squares problems posed in terms of matrices. For identifiability we insist that the elements of *D* are nonnegative. We refer to this as the ‘decomposition’ approach. Note that if we are only interested in a subset of the columns of *B*, we can replace *B* by *B*_*d*_, the *n* × *d* matrix that contains the *d* columns of interest. For notational simplicity, we suppress the subscript *d* here.

Alternatively, we can use Eq (3) as our starting point, and seek a matrix *V* with normalized columns and a diagonal matrix *D* having nonnegative entries that minimize the objective function

$$\begin{array}{c} \;\;f_{r}\left(V,D\right)=||XVD^{-1}-{B||}_{F}^{2}=\sum_{j=1}^{d_{max}}||XV_{\cdot j}D_{jj}^{-1}-B_{\cdot j}{\left|\right|}^{2} \end{array}$$ (5)

where *M*_⋅*k*_ denotes the *k*th column of *M* and where ||*C*||^2^ is the Euclidean (L^2^) norm. We refer to this as the ‘regression’ approach. Note that, unless constraints are added to the problem that mix information from the columns of *V*, the regression approach naturally separates into univariate regressions, one for each column of *B* that we are fitting. The requirement that *V* have normalized columns corresponds to *Diag*(*V*^*T*^ *V*) = *I*_*d*_.

### Unconstrained Solutions to the Decomposition and Regression Approaches

If the only constraint on *V* is that *Diag*(*V*^*T*^ *V*) = *I*, the matrices *V* and *D* that minimize Eqs (4) and (5) can be easily found. We first note a lemma governing minimizers of Eq (4):

*Lemma* 1. Let *W* minimize $f\left(W\right)=||X-BW^{T}{\left|\right|}_{F}^{2}$ where *X* has rank *q*, *B* has dimension *n* × *d* and *X* = *L*Σ*R*^*T*^ is the singular value decomposition given in Eq (1). Then *W* = *RQ* for some *q* × *d*-dimensional matrix *Q*.

The proof of Lemma 1 can be found in the appendix. Note that Lemma 1 implies that minimization of Eq (4) is equivalent to minimization of $||L\Sigma -BQ^{T}{\left|\right|}_{F}^{2}$ for *q* × *d*—dimensional matrix *Q*, which implies we can find a unique minimizer even when *p* > *n* since *q* ≤ *min*(*p*, *n*). By direct optimization we find that if the columns of *B* are orthogonal, the minimizer of Eq (4) is

$$\begin{array}{c} W_{du}≔V_{du}D_{du}=X^{T}B; \end{array}$$ (6)

given *W*_*du*_, *D*_*du*_ and *V*_*du*_ are determined by the norms of the columns of *W*_*du*_.

Unlike Eq (4), optimization of Eq (5) produces a family of solutions. The general solution can be written as

$$\begin{array}{c} Z_{ru}≔V_{ru}D_{ru}^{-1}=RQ_{ru}+R^{⊥}A_{ru} \end{array}$$ (7)

where the subscript *r* denotes regression. Using Eq (7) in Eq (5) we find

$$\begin{array}{c} Q_{ru}=\Sigma^{-}L^{T}B\;\text{.} \end{array}$$

where *M*^−^ denotes the Moore-Penrose inverse of *M*. Eq (5) gives no information on *A*_*ru*_; however, if we choose *A*_*ru*_ = 0 then Lemma 1 shows the resulting choice of *V*_*ru*_ will give the best decomposition (in the sense of minimizing Eq (4)) among all choices in the family Eq (7). Thus, we choose *A*_*ru*_ = 0, to obtain the particular solution

$$\begin{array}{c} Z_{ru}=R\Sigma^{-}L^{T}B=X^{-}B\;\text{.} \end{array}$$ (8)

As before, *V*_*ru*_ and *D*_*ru*_ are determined by the norms of *Z*_*ru*_. Note that in general *V*_*du*_ obtained by minimizing Eq (4) differs from *V*_*ru*_ obtained by minimizing Eq (5).

Because the unconstrained decomposition and regression approaches differ, it is not clear that either is adequate for our dual goal of predicting *B* for future observations and describing *X* for ordination and biplot construction. Thus, $XV_{du}D_{du}^{-1}$ may give poor prediction of *B* in the sense that Eq (5) is large, while $BD_{ru}V_{ru}^{T}$ may be a poor approximation to *X* in the sense that Eq (4) is large.

Because *V*_*ru*_ ≠ *V*_*du*_, OTUs selected as important for regression may not correspond to important variables for decomposition, or vice versa. We explore these issues further using the Tobacco data in the next section. Since both regression and decomposition are important, we next consider minimizing Eqs (4) and (5) subject to the constraint that *V* has orthonormal columns. We will see in our analysis of the tobacco data that this has the effect of ensuring that the *V* that is selected performs well for both regression and decomposition.

### Orthogonal Solutions to the Decomposition and Regression Approaches

The easy connection between Eqs (1), (2) and (3) when using measuring similarity using *XX*^*T*^ occurs because the columns of *R* are orthogonal. In order to ensure that the OTUs selected are important for both regression and decomposition, we next consider minimizing Eqs (4) or (5) subject to the constraint

$$\begin{array}{c} V^{T}V=I\;\;\;. \end{array}$$ (9)

Unless all the singular values of *X* are equal (i.e., if *X* has been standardized by right-multiplication by ${\left(X^{T}X\right)}^{-\frac{1}{2}}$), it is easy to see that neither *W*_*du*_ nor *Z*_*ru*_ have orthogonal columns. As a result, we seek *V*_*do*_ and *D*_*do*_, the minimizers of Eq (4) subject to constraint Eq (9) and *V*_*ro*_ and *D*_*ro*_, the minimizers of Eq (5) subject to constraint Eq (9), where the subscript o refers to orthogonal.

Finding *V*_*do*_ and *D*_*do*_ is related to the orthogonal but not orthonormal Procrustes problem [7]. Because the minimizer of $||X-BDV^{T}{\left|\right|}_{F}^{2}$ with respect to either *D* or *V* subject to Eq (9) is available in closed form, Everson [7] suggests the Tandem algorithm, an alternating approach in which first *V* then *D* is updated, until convergence. Finding the optimal *V* given *D* is not difficult, requiring only the calculation of a single SVD, while the optimal *D* given *V* can be expressed in closed form (see the proof of Lemma 2 in the appendix). Further, Lemma 1 implies *V* = *RQ* while Eq (9) implies *Q*^*T*^ *Q* = *I*.

Finding *V*_*ro*_ and *D*_*ro*_ is much harder, even after using Eq (7), because the closed form of *V* that minimizes the Frobenius norm subject to orthogonality constraint Eq (9) is not known even when *D* is assumed to be known. We know of three ways to numerically optimize Eq (5) subject to constraint Eq (9); none of the methods outperform the others in all cases. First, [7] gives a representation of *V* in terms of an initial matrix *V*_0_ that satisfies Eq (9) and (d2) Givens rotation matrices; this enables brute-force minimization of Eq (5) subject to Eq (9) using a derivative-free optimizer. A similar representation for the derivatives of Eq (5) w.r.t. the Givens rotation angles is possible as well. Second, an approximate quadratic programming algorithm by Watson [8], described in [7], can be used. This approach requires solving a (d2)-dimensional linear system for each step. Finally an approach described by Gower and Dijksterhuis ([9], pp98–100) using an algorithm by Koschat and Swayne [10] for finding *V* satisfying Eqs (5) and (9) for fixed *D* can be used. If only the first few columns of *V*_*ro*_ are needed, the brute force approach works well. In this situation, we can optimize Eq (5) using only *d* columns of *B*, and systematically increase *d* until the needed components stabilize. This approach assumes the first few columns of *B* explain the majority of variability; the values of *D*_*do*_ can be used as a guide to ensure that the important columns of *B* are being used.

### Decomposing The Variability in the Data Matrix *X*

Once we have obtained an estimate of *V*, it can be used either for predicting *B* (e.g., for future observations) or describing variability in *X* (e.g., for constructing biplots). Since both goals are important, we need to evaluate the performance of each method for regression and decomposition. Regression performance is easily summarized by *R*^2^, the correlation between the predicted and observed columns of *B*; note this measure is independent of *D*. Decomposition performance is a bit more complicated, since the natural quantity $||X-BDV^{T}{\left|\right|}_{F}^{2}$ depends explicitly on *D*. To avoid penalizing the regression approaches just because of the scale choice, for assessing the performance of *V*_*ro*_ in explaining variability in *X*, we replace *D*_*ro*_ by ${\tilde{D}}_{ro}$, the minimizer of Eq (4) when *V* = *V*_*ro*_. This change is unnecessary for *D*_*ru*_ since it is easy to show that it already minimizes Eq (4). We now show the following lemma that governs partitioning the total sum of squares ${\left||X|\right|}_{F}^{2}$ into a model sum of squares $||BDV^{T}{\left|\right|}_{F}^{2}$ and a residual sum of squares $||X-BDV^{T}{\left|\right|}_{F}^{2}$. If *X* is centered, then the total sum of squares is proportional to the variance of the *X*_*ij*_s.

*Lemma* 2. Let *B* be a *n* × *d*-dimensional matrix with orthonormal columns and let *D* be a *d* × *d*-dimensional diagonal matrix chosen to minimize $||X-BDV^{T}{\left|\right|}_{F}^{2}$. Then

$$\begin{array}{c} {\left||X|\right|}_{F}^{2}=||X-BDV^{T}{\left|\right|}_{F}^{2}+\;||BDV^{T}{\left|\right|}_{F}^{2}\;\; \end{array}$$

Proof of Lemma 2 can be found in the Appendix. Further, as long as *B* is orthogonal, we can decompose the model sum of squares either as

$$\begin{array}{c} ||BDV^{T}{\left|\right|}_{F}^{2}=\sum_{k=1}^{d}D_{k}^{2}\;\;\;\;\; \end{array}$$ (10)

or

$$\begin{array}{c} ||BDV^{T}{\left|\right|}_{F}^{2}=\sum_{j=1}^{J}w_{j}^{2} \end{array}$$ (11)

where $w_{j}^{2}=\sum_{k=1}^{d}W_{jk}^{2}=\sum_{k=1}^{d}{\left(V_{jk}D_{kk}\right)}^{2}$. Eq (10) partitions the model sum of squares into parts that are explained by each component, with the *k*th component contributing $D_{k}^{2}$ to the model sum of squares; Eq (11) partitions the model variability into parts explained by each OTU so that data from the *j*th OTU contributes $w_{j}^{2}$ to the model sum of squares. Thus, the value of $w_{j}^{2}/{\left||X|\right|}_{F}^{2}$ gives the proportion of the variability in ${\left||X|\right|}_{F}^{2}$ that is explained by the *j*th OTU. Using these partitions, and in particular by examining ‘scree’ plots of sorted values of $D_{i}^{2}$ or $w_{j}^{2}$, gives us another method to evaluate the performance of each method. Finally we note that Eqs (10) and (11) holds for any choice of *d*; we may wish to reserve the term ‘residual sum of squares’ for the value of $||X-BDV^{T}{\left|\right|}_{F}^{2}$ that is attained when the maximum value of *d* is used. In this case we can partition the ‘model’ sum of squares into a part corresponding to components actually used (typically, the first *d* components) and a part corresponding to the unused (truncated) components. From Eqs (10) and (11), it is easily seen that the sum of squares corresponding to truncated components can be written either as $\sum_{j=d+1}^{d_{max}}D_{j}^{2}\;$ or as $\sum_{k=d+1}^{d_{max}}W_{jk}^{2}=\sum_{k=d+1}^{d_{max}}{\left(V_{jk}D_{kk}\right)}^{2}$.

## Analysis of Bacteria found in Smokeless Tobacco Products

To illustrate the approaches developed here, we applied the decomposition and regression approaches, with and without the orthogonality constraint, to 16S rRNA data on 15 smokeless tobacco products; 6 dry snuffs, 7 moist snuffs, and 2 toombak samples from Sudan. Three separate (replicate) observations (starting with sample preparation) were made of each product, so that in total 45 observations are available. Our goal in analyzing these data are both to find important OTUs that describe the variability in the microbial communities in these products, and to develop insight on how well each approach performs in a variety of measures.

We measured distance Δ between samples using the (weighted) unifrac distance. To account for differences in read count across samples, we sub-sampled reads so that each sample had the same number of reads before calculating the distance. We repeated this subsampling 1,000 times and averaged over replicates to obtain a final matrix Δ. After centering rows and columns of the the matrix having elements $\Delta_{ij}^{2}$ as described by Gower [11], we obtained the matrix *B* by spectral decomposition of the resulting matrix. We additionally converted the rows of *X* to percent abundances to eliminate differences in scale, and then centered the rows and columns to sum to zero. All calculations were carried out using R.

The Tandem algorithm [7] applied to these data converges almost instantly even when all 44 columns of *B* are used in the decomposition. We found it much harder to find *V*_*ro*_ for all 44 components, as there are apparently local minima. The computation time was measured in hours or days, not seconds like the Tandem algorithm. A modification of the Watson [8] algorithm that used a line search to choose the step size gave the solution having the smallest value of Eq (4) that we present here.

In Table 1 we compare the performance of the four methods in terms of their ability to explain *X* and their ability to predict *B*. Results in Table 1 are based on estimating *d* = 44 components, the maximum number for these data. The most surprising result in Table 1 is the remarkably small proportion (0.8%) of the data matrix *X* that is explained by using *V*_*ru*_ chosen by unconstrained regression, even though *V*_*ru*_ predicts the columns of *B* perfectly. Although *V*_*du*_ predicts 100% of the variability in *X*, its performance in predicting the columns of *B* is the worst of the four approaches. Overall *V*_*do*_ seems to perform best, explaining almost 90% of the variability in *X* while also predicting the important columns of *B* well. The performance of *V*_*ro*_ in predicting the columns of *B* was also good but it only explained about 75% of the variability in *X*. Thus, even if prediction of *B* is the primary goal, the small improvements in *R*^2^ do not seem to warrant the computational effort required to obtain *V*_*ro*_.

**Table 1:** Percent of variation in *X* explained and *R*^2^ for prediction of *B*_⋅1_ through *B*_⋅9_ for the Tobacco data.

	% Variation of *X* Explained^1^	*R*^2^ for prediction of column:								
Analysis		1	2	3	4	5	6	7	8	9
Unconstrained Regression	0.8	1.000	1.000	1.000	1.000	1.000	1.000	1.000	1.000	1.000
Unconstrained Decomposition	100.0	0.888	0.872	0.685	0.583	0.964	0.106	0.126	0.163	0.066
Orthogonal Regression	74.5	0.974	0.885	0.896	0.910	0.981	0.808	0.751	0.693	0.622
Orthogonal Decomposition	88.9	0.962	0.909	0.845	0.818	0.979	0.789	0.741	0.682	0.398

^1^ $100|\hat{X}{|}_{F}^{2}/{\left|X\right|}_{F}^{2}$

**Fig 1:**
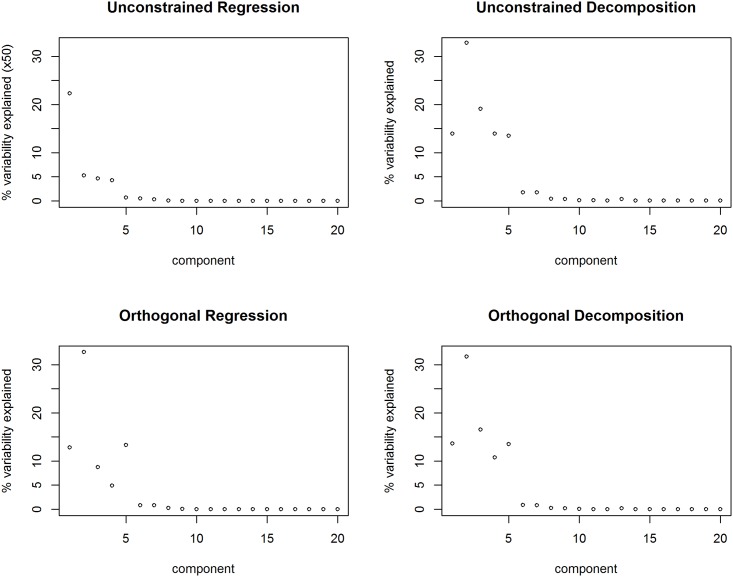
Scree plots for variance explained by each component. For each method, the contribution of the first 20 (of 44) components plotted. Note the change of scale for unconstrained regression. Components are ordered by decreasing eigenvalue of the Unifrac distance matrix.

In Fig 1 we plot the (square of the) diagonal elements of *D* for the four methods considered here: the unconstrained regression and decomposition approaches, and the orthogonal regression and decomposition approaches. For orthogonal regression we plot the (square of the) diagonal elements of ${\tilde{D}}_{ro}$ selected for the orthogonal regression approach when used as a decomposition method. Like a typical SVD, the ‘scree plot’ shows that for each decomposition only a few components are important. This is reassuring as we are most interested in truncated versions of the SVD-like decompositions. From Fig 1 we are assured that a biplot in 2 or 3 dimensions will capture much of the variability in the data. Note that the components are sorted by the eigenvalues of *B*, not the magnitude of *D*, so that the generally monotonic decrease in *D* values indicates directions that are important in describing *B* are also important in describing *X*. If this were not the case, it may be worth choosing another measure of distance for calculating *B*.

**Fig 2:**
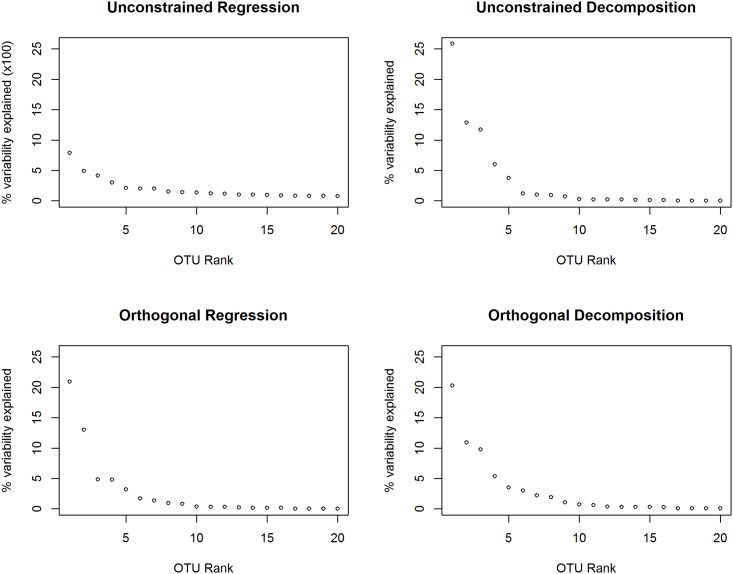
Scree plots for variance explained by each OTU. For each method, the contribution of the 20 (of 271) OTUs having the largest contributions are plotted. Note the change of scale for unconstrained regression.

In Fig 2 we plot the sorted values of $w_{j}^{2}$ for each method considered here; note that the order of OTUs may be different in each panel. To see how similar the orderings OTU influence (as measured by $w_{j}^{2}$) are across methods, we calculated the variance-covariance matrix of the $w_{j}^{2}$ values from the four approaches (Table 2). These correlations are high except when calculating $w_{j}^{2}$ using unconstrained regression, indicating that the ordering of OTUs in Fig 2 is similar for all the methods except unconstrained regression. Since Tyx et al. [6] found 3 principal components were necessary to separate these three groups, we used *d* = 3 when calculating $w_{j}^{2}$. In Table 3 we show the 11 OTUs that were selected to be on the list of the top 5 OTUs for each method (along with the variability explained by that OTU and its rank by each method). There is good agreement between both decomposition approaches and the orthogonal regression approach, while none of the OTUs selected by unconstrained regression appear on the top 5 list for any other method. The OTUs selected by unconstrained regression are biologically distant as well, with only one OTU selected by unconstrained regression sharing a family (*Staphylococcaceae*) with any OTU selected by one of the other methods.

**Table 2:** Correlation between $w_{j}^{2}$ values for each method.

	Unconstrained Regression	Unconstrained Decomposition	Orthogonal Regression	Orthogonal Decomposition
Unconstrained Regression	1	−0.01	−0.01	−0.01
Unconstrained Decomposition		1	0.95	0.99
Orthogonal Regression			1	0.96

**Table 3:** Taxonomic assignment for OTUs selected as a top 5 OTU in explaining variability, and the variability explained by each OTU (*d* = 3 components).

				Unconstrained Regression		Unconstrained Decomposition		Orthogonal Regression		Orthogonal Decomposition	
OTU^1^	Family	Genus	Species	Rank	VE^1^	Rank	VE	Rank	VE	Rank	VE
29012	*Enterococcaceae*	*Tetragenococcus*	*halophilus*	59	2.3 × 10^−5^	4	6.0 × 10^−2^	4	4.8 × 10^−2^	4	5.3 × 10^−2^
52399	*Aerococcaceae*	*Unknown*	*unknown*	58	2.3 × 10^−5^	3	0.12	2	0.13	2	0.11
181589	*Staphylococcaceae*	*Staphylococcus*	*equorum*	1	7.9 × 10^−4^	30	1.3 × 10^−4^	32	1.3 × 10^−4^	28	2.3 × 10^−4^
801438	*Enterobacteriaceae*	*Unknown*	*unknown*	5	2.1 × 10^−4^	55	2.4 × 10^−5^	58	2.3 × 10^−5^	55	4.2 × 10^−5^
810425	*Corynebacteriaceae*	*Corynebacterium*	*unknown*	103	6.4 × 10^−6^	6	1.2 × 10^−2^	6	1.7 × 10^−2^	5	3.5 × 10^−2^
905303	*Aerococcaceae*	*Alloiococcus*	*unknown*	57	2.4 × 10^−5^	5	3.7 × 10^−2^	3	4.9 × 10^−2^	6	3.0 × 10^−2^
1102921	*Carnobacteriaceae*	*Granulicatella*	*unknown*	2	4.9 × 10^−4^	56	2.3 × 10^−5^	62	2.0 × 10^−5^	64	2.2 × 10^−5^
1110381	*Aerococcaceae*	*Unknown*	*unknown*	4	3.0 × 10^−4^	153	6.0 × 10^−7^	171	7.0 × 10^−7^	171	4.4 × 10^−7^
4297253	*Bacillaceae*	*Bacillus*	*unknown*	3	4.2 × 10^−4^	20	3.3 × 10^−4^	17	4.5 × 10^−4^	20	7.2 × 10^−4^
4312974	*Staphylococcaceae*	*Staphylococcus*	*succinus*	69	1.7 × 10^−5^	1	0.26	1	0.21	1	0.20
4379247	*Lactobacillaceae*	*Lactobacillus*	*unknown*	76	1.5 × 10^−5^	2	0.13	5	3.2 × 10^−2^	3	9.8 × 10^−2^

^1^Greengenes OTU (operational taxonomic unit) Identification Number, VE = Variance Explained $w_{j}^{2}/{\left|X\right|}_{F}^{2}$

**Fig 3:**
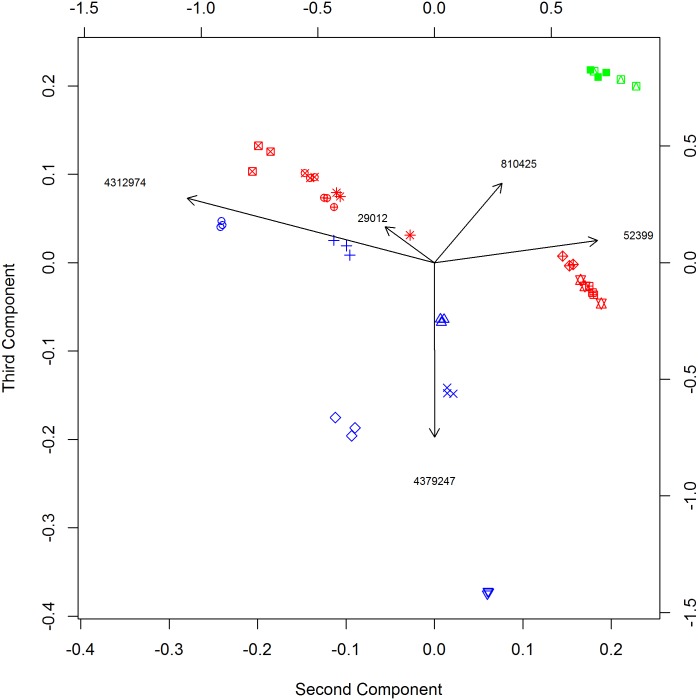
Biplot for second and third component of tobacco bacterial data. Points are colored by type (blue = dry, red = moist, green = toombak) and samples corresponding to replicates of the same product are plotted with the same symbol. The taxonomic families corresponding to the OTUs shown are *Staphylococcaceae* (4312974), *Aerococcaceae* (52399), *Lactobacillaceae* (4379247), *Enterococcaceae* (29012) and *Corynebacteriaceae* (810425). The scale on bottom and left corresponds to coordinates of samples, scale on right and top to coordinates of OTUs.

The effect of each OTU can be displayed in a biplot. In Fig 3 we show a 2-dimensional biplot based on the orthogonal decomposition method, showing the second and third components (which had the two highest values of both *D*_*du*_ and *D*_*do*_). It is clear the ordination of these data using the first 3 PCs of the (weighted) Unifrac matrix are fairly successful at separating the different types (dry, moist and toombak). Further, the replicates corresponding to the same product are tightly clustered. We also show arrows corresponding to the top five OTUs calculated using orthogonal decomposition. To construct this biplot, we note that the orthogonal decomposition implies

$$\begin{array}{c} X_{ij}\approx \sum_{k}B_{ik}D_{kk}V_{jk}=\ll B_{i},W_{j}\gg \; \end{array}$$ (12)

where *B*_*i*_ denotes the *i*th row of *B* and *W*_*j*_ denotes the *j*th column of *W* = *V* ⋅ *D* and ≪*A*, *B*≫ denotes the Euclidean inner product. Since the elements of *B*_*i*_ are the coordinates of the *i*th observation and *W*_*j*_ is the vector whose norm determines the influence of OTU *j* in explaining the model sum of squares, it is natural to represent OTUs by plotting *W*_*j*_. Further, the magnitude of *X*_*ij*_ is represented by the dot product of *B*_*i*_ and *W*_*j*_, so that if *W*_*j*_ for an OTU ‘points towards’ a certain group of samples, we can expect that the values of *X*_*ij*_ are relatively large for these samples. To create a low-dimensional plot, we typically sum *k* in Eq (12) over two or three dimensions; for Fig 3 we sum *k* from 2 to 3.

By examining the biplot in Fig 3, we see that Sudanese toombak is characterized by elevated levels of OTU 810425, assigned to the Corynebacteriaceae family, largely absent from all other types. The OTU 4379247 (Lactobacillaceae) appears elevated in some dry snuff samples; whereas, OTUs 29012 (Enterococcaceae), 4312974 (Staphylococcaceae) and 52399 (Aerococcaceae) appear elevated in moist snuff samples.

## Additional Considerations

In this section, we show that the results we have obtained can be applied directly to some simple but important generalizations. In particular, we show how to incorporate rarefaction into our decomposition approach, and indicate where it may not be necessary. We also consider a weighted regression approach that gives a connection between the regression and decomposition approaches.

Rarefaction is a commonly used (but still controversial, see e.g. [12]) approach to processing microbiome data to account for differences in library size. In our analysis of the tobacco data, we averaged over rarefactions when calculating the distance matrix; here we address the question of how to incorporate averaging over rarefactions of the data matrix into our orthogonal decomposition. Computing a separate decomposition for each rarefaction is not tenable as it is unclear how we would combine the decompositions obtained for each replicate. Instead, we propose finding *D* and *V* that minimize the objective function

$$\begin{array}{c} f_{dR}\left(D,V\right)=\frac{1}{R}\sum_{r=1}^{R}\left|\right|X_{r}-BDV^{T}{\left|\right|}_{F}^{2}\; \end{array}$$

subject to *D* ≥ 0 and the desired constraints on *V*, where *X*_*r*_ is the *r*^th^ rarefied data matrix. However, since

$$\begin{array}{ll} ||X_{r}-BDV^{T}|{|}_{F}^{2} & =Tr\left(X_{r}^{T}X_{r}\right)-2Tr\left(X_{r}^{T}BDV^{T}\right)+Tr\left(VDB^{T}BDV^{T}\right)\\ & \begin{array}{c} =C-2Tr\left(X_{r}^{T}BDV^{T}\right)+Tr\left(D^{2}\right), \end{array} \end{array}$$

we see that we can instead optimize $\left|\right|\bar{X}-BDV^{T}\left|\right|$ where $\bar{X}$ is the average of the data matrix over rarefactions. Thus, if *X* contains the untransformed counts (or even if the data matrix is scaled by the library size for each observation), in the limit this corresponds to using ${\bar{X}}_{ij}=\pi_{ij}M$ (or ${\bar{X}}_{ij}=\pi_{ij}$ if we scale the rows of *X* by the library sizes), where *π*_*ij*_ is the frequency of the *j*th OTU in the *i*th sample and *M* is the number of reads selected in each rarefaction. Since centering for PCoA is also linear in the elements of *X*, this argument suggests that using the empirical frequencies without rarefaction, at least for the decomposition approaches, is warranted.

Turning now to the relationship between the orthogonal regression and decomposition approaches, the objective function for the orthogonal regression approach given in Eq (5) assigns equal importance (weight) to the prediction of each column of *B*. If we choose to weight the prediction of the *j*th column of *B* by $D_{jj}^{2}$, a measure of the importance of the *j*th column, then it is easy to show that minimizing the resulting objective function $\left|\right|\left(XVD^{-1}-B\right){D||}_{F}^{2}=||XV-{BD||}_{F}^{2}$ yields the same values of *V* and *D* as minimizing the objective function for orthogonal decomposition. Thus, orthogonal decomposition also has an interpretation as a weighted regression, where the weight assigned to the prediction of each column is proportional to the variance of *X* explained by that column in the decomposition.

## Discussion

The principal components of a distance matrix Δ can be very useful in ordination, the representation of observations in an Ecology or microbiome study as points in a low-dimensional space. Meaningful groupings in the data are often apparent in an ordination plot. When the correlation matrix is used to measure similarity, there is a natural duality that enables us to express the eigenvectors of Δ as linear combinations of the species or OTU frequencies. This duality allows construction of a biplot, in which both observations and OTUs can be simultaneously represented graphically. When an arbitrary distance is used, we have developed methods to restore this duality, at least approximately. We evaluated these approaches within the context of an analysis of the bacterial species found in smokeless tobacco products [6].

In our analysis of the bacteria found in smokeless tobacco products, we found that the orthogonality constraint results in linear combinations that perform well both in explaining the variability in the data matrix *X* as well as predictors in a regression. This is reasonable as orthogonality is the same principle connecting the regression and decomposition approaches in a SVD of *X*. We also found that the orthogonal regression and orthogonal decomposition approaches gave similar results, which were also fairly close to the unconstrained decomposition approach. Finally, given the difficulties in obtaining more than a few components of the orthogonal regression approach, and the interpretation of the orthogonal decomposition approach as a weighted version of the orthogonal regression approach given in the previous section, it seems that the orthogonal decomposition approach is the most appealing approach.

We also showed that the approaches we presented have a variance partitioning property in which the total sum of squares represented by ${\left||X|\right|}_{F}^{2}$ can be partitioned into residual sums of squares and model sums of squares. We further showed that, even when we choose a set of linear combinations *V* that are not orthogonal, the model sum of squares can be partitioned in two ways; one in which we sum over the contributions of each component, another in which we sum over the contributions of each variable (OTU). The first partition can be used to justify a truncated decomposition; the second can be used to find important variables, especially for making biplots. We found that both orthogonal approaches and unconstrained decomposition were in broad agreement (similar model sums of squares, similar OTUs identified as important) while unconstrained regression behaved very differently, identifying very different OTUs as important and having a small model sum of squares. This may be because a certain set of OTUs may allow good prediction of a columns of *B* even if these OTUs do not explain much of the overall variability in the OTU table (e.g., if they are rare). Since the decomposition approaches also give good prediction of at least those columns of *B* that explain most of the variability (at least in the tobacco data we considered) it seems that unconstrained regression can miss important large-scale features in favor of small-scale features that happen to be good predictors of *B*, in some sense failing to see the forest through the trees.

We have considered here only decompositions of the data matrix *X*. The results here thus can be considered ‘unsupervised learning.’ In further work, we plan to consider extensions of this approach to ‘supervised learning’ where we have additional variables that we wish to incorporate into the choice of linear combinations. For example, we may wish to find linear combinations of OTUs that optimally explain group membership (e.g., tobacco type in the tobacco data considered here).

## Appendix: Proofs of the Lemmas

Proof of Lemma 1. Because *q* is the rank of *X*, *p* ≥ *q*. If *p* = *q* the result is trivial, since the columns of *R* span $R^{p}$ and so the columns of any *p* × *d*-dimensional matrix *W* can be expressed as a linear combination of columns of *R*, which establishes the result. For *p* > *q* let *R*^⊥^ be a (*p* − *q*) × *p*-dimensional matrix having orthonormal columns that span the orthogonal compliment of the space spanned by the columns of *R*. Because the columns of any *p* × *d*-dimensional matrix can we written in terms of the basis given by the columns of *R* and *R*^⊥^ we have *W* = *RQ* + *R*^⊥^ *A* for matrices *Q* and *A*. Inserting this form into *f*(*W*) and using *R*^*T*^ *R*^⊥^ = 0 and *XR*^⊥^ = 0 we find $f\left(W\right)=||X-BQ^{T}R^{T}{\left|\right|}_{F}^{2}+\left|\right|A^{T}A{\left|\right|}_{F}^{2}$. Since *A*^*T*^ *A* is a real symmetric matrix, *f*(*W*) is minimized when *A* = 0, i.e. when *W* = *QR*.

Proof of Lemma 2: By direct calculation, each element *D*_*jj*_ satisfies (*B*^*T*^ *XV*)_*jj*_ = *Djj*(*V*^*T*^ *V*)_*jj*_. The lemma holds because $Tr\left[VDB^{T}\left(X-BDV^{T}\right)\right]=Tr\left(DB^{T}XV-D^{2}V^{T}V\right)\;=\sum_{j=1}^{d}D_{jj}{\left(B^{T}DV\right)}_{jj}-D_{jj}^{2}{\left(V^{T}V\right)}_{jj}=0$ elementwise. Finally, note if *D*_*jj*_ < 0 we can replace *D*_*jj*_ by −*D*_*jj*_ while replacing *V*_*kj*_ by −*V*_*kj*_ ∀*k* and the lemma still holds.

## References

[1] Caporaso JG, Kuczynski J, Stombaugh J, Bittinger K, Bushman FD, Costello EK, et al QIIME allows analysis of high-throughput community sequencing data. Nat Meth. 2010 5;7(5):335–336. 10.1038/nmeth.f.303 PMC3156573 20383131

[2] Schloss PD, Westcott SL, Ryabin T, Hall JR, Hartmann M, Hollister EB. Introducing mothur: Open-source, platform-independent, community-supported software for describing and comparing microbial communities. Appl Environ Microbiol 2009 12;75(23):7537–41. 10.1128/AEM.01541-09 19801464 PMC2786419

[3] Lozupone CA, Hamady M, Kelley ST, Knight R. Quantitative and qualitative beta diversity measures lead to different insights into factors that structure microbial communities. Appl Environ Microbiol 2007 3;73(5):1576–85. 10.1128/AEM.01996-06 17220268 PMC1828774

[4] Lozupone C, Knight R. UniFrac: A New Phylogenetic Method for Comparing Microbial Communities. Appl Environ Microbiol 2005 12;71(12):8228–8235. 10.1128/AEM.71.12.8228-8235.2005 16332807 PMC1317376

[5] Legendre P, Legendre L. Numerical Ecology. 3rd English Edition Amsterdam: Elsevier; 2012.

[6] Tyx RE, Stanfill SB, Keong LM, Rivera AJ, Satten GA Watson CH. Characterization of bacterial communities in selected smokeless tobacco products. PLoS ONE 2016 1;11(1):e0146939 10.1371/journal.pone.0146939 26784944 PMC4718623

[7] Everson R. Orthogonal, but not Orthonormal, Procrustes Problems; 1998. Available from: http://secamlocal.ex.ac.uk/people/staff/reverson/uploads/Site/procrustes.pdf

[8] Watson G. The solution of orthogonal Procrustes problems for a family of invariant norms. Adv Comput Math 1994 9;2(4):393–405. 10.1007/BF02521606

[9] Gower JC, Dijksterhuis GB Procrustes Problems. New York: Oxford University Press; 2004

[10] Koschat MA, Swayne DF A weighted Procrustes criterion. Psychometrika 1991 6;56(2):229–239. 10.1007/BF02294460

[11] Gower JC Some distance properties of latent root and vector methods used in multivariate analysis. Biometrika 1966 12;53(3 and 4):325–338. 10.2307/2333639

[12] McMurdie PJ, Holmes S. Waste not, want not: Rarefying microbiome data is inadmissible. Plos Comp Biol 4 2014;10(4):e1003531 10.1371/journal.pcbi.1003531 PMC3974642 24699258

